# Flavour Compensation Role of Yeast Strains in Reduced-Salt Dry Sausages: Taste and Odour Profiles

**DOI:** 10.3390/foods11050650

**Published:** 2022-02-23

**Authors:** Xiang-Ao Li, Baohua Kong, Rongxin Wen, Huiping Wang, Mengtong Li, Qian Chen

**Affiliations:** College of Food Science, Northeast Agricultural University, Harbin 150030, China; lixiangao67@163.com (X.-A.L.); kongbh63@hotmail.com (B.K.); wenrongxin810@163.com (R.W.); whp_1998@163.com (H.W.); limengtong0923@163.com (M.L.)

**Keywords:** dry sausage, salt reduction, yeast inoculation, flavour compensation, taste profile

## Abstract

The effects of different yeast strains including *Pichia kudriavzevii*, *Torulaspora delbrueckii*, and *Debaryomyces hansenii* on the taste and odour profiles of reduced-salt dry sausages were explored. Inoculation of *P. kudriavzevii* and *D. hansenii* compensated for the lack of saltiness and umami tastes of reduced-salt sausages. Furthermore, inoculation of *P. kudriavzevii* and *T. delbrueckii* resulted in an odour profile in the reduced-salt dry sausages that was similar to traditional dry sausages. According to the volatile analysis, the contents of certain alcohols, acids, esters and terpenes were higher in the inoculated sausages. Finally, the sensory evaluation indicated that the inoculation of *P. kudriavzevii* and *D. hansenii* contributed positively to the aroma and saltiness of reduced-salt dry sausages. In conclusion, *P. kudriavzevii* and *D. hansenii* can be employed as effective starter cultures to compensate for the flavour deficiencies of reduced-salt dry sausages.

## 1. Introduction

Salt (sodium chloride, NaCl), an essential ingredient in meat products, plays a key role in providing saltiness, enhancing umami, creating desired textures, and prolonging shelf life [1,2,3]. Essential salt intake is required for normal body functions, however, excessive salt intake may increase the risk of hypertension, as well as cardiovascular, renal, and bone diseases [4,5]. Most people reportedly consume an average of 9–12 g of salt per day [6], which exceeds the recommended adult NaCl consumption limit (<5 g/day) established by the World Health Organization (WHO) [7]. Dry-cured meat products, especially dry sausages and dry-cured hams are among the primary sources of sodium intake, accounting for approximately 20% of the total intake [8]. In general, 2.5% of NaCl is added to dry sausages during their preparation. However, this level increases to 3.6–5% after dehydration during fermentation [9]. Therefore, low-sodium dry sausages have recently garnered increasing attention [10,11,12].

Several strategies have been proposed for reducing the sodium content of meat products, such as directly reducing NaCl addition levels, replacing NaCl with chloride salts, and changing the physical structure of NaCl, among others [13]. Among these, directly reducing NaCl addition is the most practical approach for both merchants and consumers, as it is less costly and avoids the undesirable flavours caused by chloride salts [14]. However, despite effectively reducing sodium intake, this strategy also negatively impacts the palatability and quality characteristics of meat products.

The application of appropriate starter cultures may be an alternative solution for reducing the salt level of fermented products, which can stabilize and standardize the fermentation process, enhance quality, and improve the safety of the final products [15]. Lactic acid bacteria (LAB) together with coagulase-negative staphylococci are the most common bacterial starter cultures in fermented meat products due to their contribution to the acidification process, colour development, and flavour formation [16]. Additionally, some fungi, such as yeasts also play a crucial role in fermented meat products. The yeast genera most frequently found in fermented meat products include *Debaryomyces*, *Candida*, *Trichosporon*, *Pichia*, *Rhodotorula*, *Yarrowia*, and *Cryptococcus* [17]. The quality of dry sausages can be enhanced through increasing pH via lactic acid utilization by yeast metabolism, stabilising the cured colour via antioxidant enzymes of yeasts [18,19], and promoting the flavour formation via the esterase and lipolytic and proteolytic enzymes of yeasts [20]. Further, yeast inoculation prevents the proliferation of harmful microorganisms, as well as mycotoxin accumulation [21]. Therefore, yeast strains could potentially compensate for the quality defects of reduced-salt dry sausages.

The flavour profile of dry sausages, including taste and odour properties, is a crucial indicator of their sensory characteristics. Free amino acids (FAAs), peptides, and organic acids, which originate from proteolysis and carbohydrate metabolism, play an important role in the taste and odour development of meat products [22]. Particularly, some FAAs and peptides provide saltiness and umami, and thus, may enhance the taste properties of reduced-salt dry sausages. In addition, the taste compensation role of some LAB strains has been confirmed in reduced-salt dry sausages [23]. Therefore, we hypothesised that yeasts could compensate for the flavour deficiencies of reduced-salt dry sausages.

In our previous study, 46 yeast strains were isolated from traditional dry sausages collected from Northeast China [24]. This type of dry sausage is a small calibre sausage subjected to a fast fermented process (approximately 12 days) and can be consumed after cooking. After evaluation, three strains (*Pichia kudriavzevii*, *Torulaspora delbrueckii*, and *Debaryomyces hansenii*) with excellent fermentation capabilities and flavour formation potential were selected for further characterization [25]. The effects of yeast inoculation on the physical, microbial, and quality characteristics of salt-reduced dry sausages were then investigated. The taste and odour properties of the resulting sausages were analysed via electronic tongue, electronic nose, and headspace solid-phase microextraction/gas chromatography-mass spectrometry (HS-SPME/GC-MS) in combination with partial least squares-discriminant analysis (PLS-DA) to evaluate the flavour compensation role of these yeast strains. This work will provide excellent starter cultures that can improve the quality and flavour characteristics of reduced-salt dry sausages.

## 2. Materials and Methods

### 2.1. Preparation of Yeast Strains

The flavour-enhancing properties of *Pichia kudriavzevii*, *Torulaspora delbrueckii*, and *Debaryomyces hansenii* were characterised in this study. These yeast strains were isolated from traditional dry sausages and identified via ITS (internal transcribed spacer) rRNA sequencing. All of them have been shown to be well resistant to environmental stress (pH, sodium chloride and sodium nitrite) and have good flavour formation potential (proteolytic and lipolytic activities) in a fermented sausage model system. All yeast strains were inoculated (2%, *v*/*v*) into fresh YPD (yeast extract peptone dextrose) medium and cultured for 24 h at 28 °C. After incubation, the yeast cultures were centrifuged at 6000× *g* for 10 min at 4 °C to collect cells. The cells were then washed twice with sterile saline solution and resuspended in 15 mL of sterile saline solution before use.

### 2.2. Preparation of Dry Sausages

Dry sausages were prepared as described by Hu et al. [10] with slight modifications. The sausages were manufactured with lean pork and pork back fat minced through a 1.5 cm plate with the following additives: salt, sodium nitrite, monosodium glutamate, dextrose, wine, ginger powder, and mixed spices. The mixed spices included fennel, nutmeg, cloves, cassia bark, angelica, orange peel, and *Amomum villosum*. The traditional dry sausage with 2.5% NaCl (CT) and the reduced-salt dry sausage with 1.75% NaCl (CR) were regarded as the control treatments for spontaneous fermentation. Three treatments with 1.75% NaCl were inoculated with one of three starter cultures: *P. kudriavzevii* (PK), *T. delbrueckii* (TD), or *D. hansenii* (DH). Each strain was inoculated in the minced meat and its inoculation level was approximately 10^6^ CFU/g. The recipes and starter cultures are shown in Table 1.

The meat was mixed with the ingredients and filled into natural porcine casings until each fresh sausage was approximately 20 cm long with a 2.5 cm diameter. The sausages were then allowed to air-dry naturally at 25 ± 2 °C (30–50% relative humidity) for one day, then transferred to an incubator with the same temperature (65–75% relative humidity) for eleven days. Three independent batches of dry sausages (replicates) were prepared, and 60 sausages were prepared in each batch. The raw sausages were subjected to physical, microbial, colour, and flavour analysis, and the cooked sausages were subjected to shear force analysis and sensory evaluation. Finally, 15 sausages were collected on days 0, 4, 8, and 12 for each time to measure various indicators immediately.

### 2.3. Analysis of Physical, Microbial, and Quality Characteristics of Dry Sausages

The sausages were minced in a grinder (HX-J3063, AUX, Ningbo, China) for moisture content and pH measurement. Moisture content was measured via the AOAC method [26] and pH was measured according to Hu et al. [27]. LAB and yeast counts were determined according to Wen et al. [28] and Bolumar et al. [29] with slight modifications. Five grams of sausages were homogenised in 45 mL of sterile saline and decimal dilutions were prepared in the same solution. LAB counts were determined in de Man–Rogosa–Sharpe (MRS) agar after incubation at 37 °C for 2 days. Yeast counts were determined in YPD agar at 28 °C for 2 days. Shear force was determined using a texture analyser (Stable Micro Systems; TA-XT PLUS, Godalming, UK) coupled with the shear blade (Warner-Bratzler G146, Los Angeles, CA, USA) according to the method of Hu et al. [30]. The sausages were cooked at 90 °C for 20 min (core temperature 74 °C) and cooled to room temperature (25 °C), then were cut at least six cylinders of 20 mm thickness for each treatment. The shear force analysis parameters were set as follows: force sensor (30 kg), pre-test speed 2 mm/s, test speed 2 mm/s, post-test speed 2 mm/s, distance 8 mm, trigger force 30 g. The colour of sausages, characterised by the L*-value, a*-value, and b*-value, was determined by a ZE-6000 colourimeter (Nippon Denshoku, Kogyo Co., Tokyo, Japan) with a 10° observer and a D65 light source. The sausages were sliced, and the colour was measured on the cut surface in three different points of each sample. A white standard plate (L* = 95.26, a* = −0.89, b* = 1.18) was used for calibration prior to measurement [31].

### 2.4. Electronic Nose Analysis

The electronic nose analysis was performed using a PEN3 E-nose (Airsense Analytics GmbH, Schwerin, Germany) as described by Zhang et al. [32] with slight modifications. Odours can be distinguished by ten sensors that generate responses based on the different chemical properties of volatiles, including W1C, W5S, W3C, W6S, W5C, W1S, W1W, W2S, W2W, and W3S sensors and the information on these sensors is shown in Table 2. Minced dry sausage samples (3 g) were placed in a 20-mL headspace vial and incubated for 45 min at 50 °C. The electronic nose parameters were set as follows: chamber flow rate, 200 mL/min; injection flow rate, 200 mL/min; measurement duration, 120 s. The sealed headspace bottle was punctured with a needle to absorb the volatiles from the headspace at a constant rate. The sensor array was purged with pure air as a carrier gas to return the signal response to zero.

### 2.5. Electronic Tongue Analysis

The taste profiles of dry sausages were analysed by a TS-5000Z electronic tongue (Insent Company, Atsugi-Shi, Japan) with a wide-range selection of specific artificial lipid membrane sensors. Electronic tongue systems contain five chemical sensors: CA0 (sourness sensor), AE1 (astringency sensor), AAE (umami sensor), CT0 (saltiness sensor), and C00 (bitter sensor). For the analysis, 30 g of minced sausage samples were mixed with 150 mL of distilled water to extract the taste substances. The mixture was placed in a water bath at 40 °C for 30 min, then centrifuged at 5000× *g* for 10 min. The electronic tongue was adjusted and calibrated according to the manufacturer’s instructions before conducting the measurements at room temperature. The sensors were washed with distilled water for 30 s between each measurement to ensure the stability and accuracy of the results. Additionally, the sensors were checked to ensure that they were operating in the correct mV range before each measurement. The sensor was immersed in the sample solution for 30 s and six replicate measurements were performed on each sample.

### 2.6. Volatile Compound Analysis

The volatile compounds in dry sausages were extracted via an HS-SPME device (Supelco, Bellefonte, PA, USA) and analysed via GC-MS (GCMS-QP 2020 NX, Shimadzu Co., Kyoto, Japan) as described by Wen et al. [9]. A total of 3 g of minced sausage and 2 μL of 1,2-dichlorobenzene (100 mg/L in methanol), which was used as the internal standard (IS), were mixed in a 20-mL headspace vial (CNW Technologies, Duesseldorf, Germany) at 45 °C for 25 min to equilibrate and then at 45 °C for 30 min to be extracted with a polydimethylsiloxane/divinylbenzene/carboxen (PDMS/DVB/CAR)-coated SPME fibre. The volatiles in the fibre were then released by thermally desorbing at 230 °C for 3 min in the GC injector and analysed via GC-MS. The running program was as follows, GC oven was maintained at 40 °C for 3 min, and then it raised to 200 °C at a degree of 5 °C/min, and finally heated to 230 °C at 10 °C/min to be kept for 4 min. The ion source temperature was 230 °C and the mass spectrometer scanned masses were from m/z 45 to 500. Volatile compounds were determined by comparison with the NIST 17 experimental mass spectra library and those reported in the Flavornet database (http://www.flavornet.org/flavornet.html, accessed on 4 June 2021). Similarities greater than 90% were taken as identification results. Volatiles were semi-quantified by dividing the peak area of the target compounds by the peak area of the IS and multiplying this ratio by the initial concentration of the IS (expressed in µg/kg). The linear retention index (LRI) of the volatiles was determined by calculating the retention index relative to a series of standard alkanes (C9–C33).

### 2.7. Sensory Evaluation

Sensory evaluation was performed as described by Chen et al. [33] with some modifications. The sensory assessment team consisted of 20 faculty and graduate students (10 males and 10 females) with experience in sensory evaluation of fermented meat products. All panellists participated in discussions to clarify the reference for the quantitative descriptive analysis on colour, hardness, aroma, and taste attributes and were trained by these criterions. Taste attribute standards were prepared for the panellists to familiarize the taste attributes, including lactic acid (10 mM), sodium chloride (12 mM) and monosodium glutamate (4 mM). The intensity or degree of each attribute was expressed by a seven-point descriptive scale from 1 (low intensity) to 7 (high intensity). Colour is defined as the intensity of red on the surface of the dry sausage slice (1 = light red; 7 = dark red). Hardness is defined as the force required to compress the sausages with the molars (1 = extremely soft; 7 = extremely hard). Aroma is defined as the intensity of the characteristic odour of the sausages (1 = weak dry sausage aroma; 7 = strong dry sausage aroma). Sourness is defined as the typical taste of citric acid (1 = light acid taste; 7 = strong acid taste). Saltiness is defined as the typical taste of sodium chloride (1 = light salty taste; 7 = strong salty taste). Umami is defined as the typical taste of monosodium glutamate (1 = light umami; 7 = strong umami). All sausages fermented for 12 days were cut into small 2 mm thick slices after cooking at 90 °C for 20 min (core temperature 74 °C) and served randomly on white plastic plates that were randomly coded with four digits. Prior to scoring, a “warm-up” sample was provided to prompt the panellists of the scoring range for each sensory attribute. Each panellist was required to take a 1 min break and provided with purified water to clear their palates while tasting different samples.

### 2.8. Statistical Analysis

Three independent batches of dry sausages (replicates) were manufactured, and five treatments in each batch were prepared. Each experiment except for shear force analysis was conducted three times (triplicate observations). The data were analysed using the General Linear Model procedure in the Statistix 8.1 software package (Analytical Software, St. Paul, MN, USA) and the results were expressed as the mean values ± standard errors (SE). Analysis of variance (ANOVA) coupled with Tukey’s multiple comparison test was used in this study to identify significant differences between samples (*p* < 0.05). Variation in physical, microbial, and quality characteristics were described via a mixed model, with the different treatments and fermentation times as fixed terms, and each replicate as a random term. For the electronic nose, electronic tongue, and volatile compound analysis, fixed terms for a mixed model included different treatments and fermentation times, and a random term was each replicate. For sensory evaluation, a fixed term for a mixed model included different treatments, and random terms included sausage and sensory panel (session number, tasting order and panellist number). Principal component analysis (PCA) was used to illustrate the relationship between the response values of the electronic nose and tongue analyses and their respective sensors using the Origin 2019 software (OriginLab Corporation, Northampton, MA, USA). PLS-DA of the volatile compounds was performed by an online platform called MetaboAnalyst 5.0 (https://www.metaboanalyst.ca/, accessed on 13 December 2021). The variable importance in the projection (VIP) values were calculated based on the supervised PLS-DA model to determine the key differential volatile compounds.

## 3. Results and Discussion

### 3.1. Physical, Microbial, and Quality Characteristic Analysis

As depicted in Table 3, the moisture content in all dry sausages decreased significantly during fermentation (*p* < 0.05). After 12 days of fermentation, the moisture content decreased from approximately 70.73% to 18.35%, 19.36%, 19.79%, 19.85%, and 21.56% for the CT, CR, PK, TD, and DH treatments, respectively. The CT treatment exhibited a lower moisture content than the CR treatment, which was due to the different salt concentrations. The higher salt concentration may cause higher moisture evaporation [34]. The treatment inoculated with *D. hansenii* had the highest moisture content among all inoculated treatments on day 12 (*p* < 0.05). This phenomenon of delayed moisture content reduction was previously reported by Perea-Sanz et al. [35] who reported that the inoculation of *D. hansenii* could significantly increase the moisture content at the end of fermentation. Nevertheless, this phenomenon was not observed in the PK and TD treatments in our experiments, which may be due to the better ability of *D. hansenii* to accumulate Na^+^ and maintain cation homeostasis to avoid the osmotic shock induced by a large amount of salt [25]. After 8 days of fermentation, the pH of dry sausages gradually decreased from approximately 6.09 to 5.59, 5.40, 5.16, 5.07, and 5.31 for the CT, CR, PK, TD, and DH treatments (*p* < 0.05). This may be due to the consumption of carbohydrates by the lactic acid bacteria, resulting in the accumulation of lactic acid. Moreover, on day 8, the pH of inoculated sausages was lower than those of the two controls, which may be because yeast inoculation makes carbohydrates easier to metabolise by lactic acid bacteria [8]. The pH of all sausages was steady from day 8 to day 12, which may have been from the NH_3_, amines, and alkaline compounds produced by protein hydrolysis [36] and the decomposition of organic acids metabolised by yeast and fungi [37].

The yeast counts in the CT, CR, and TD treatments significantly increased from day 0 to day 8 (*p* < 0.05), and the PK and DH treatments significantly increased from day 0 to day 4 (*p* < 0.05). The LAB counts in the CT, CR, TD, and DH treatments significantly increased from day 0 to day 8 (*p* < 0.05), and the PK treatment significantly increased from day 0 to day 4 (*p* < 0.05). After reaching the maximum value, microbial counts decreased significantly (*p* < 0.05) in all treatments except for yeast counts in the CR treatment and LAB counts in the CT treatment. This may be attributed to the suitable environment at the early stage of fermentation; however, nutrient consumption and low moisture content continued as fermentation progressed. After 12 days of fermentation, there was no significant difference in the yeast count among the inoculated treatments (*p* > 0.05); however, yeast inoculation affected LAB growth. The LAB counts in the PK, TD, and DH treatments reached 7.56, 7.20, and 7.56 log CFU/g, respectively, whereas the CR treatment with the same salt content was 7.33 log CFU/g. Different yeast species are known to interact differently with LAB [37]. In this study, *T. delbrueckii* inhibited LAB growth whereas *P. kudriavzevii* and *D. hansenii* promoted LAB growth.

The decreasing moisture content described above may be responsible for the significant increase in shear force during fermentation (*p* < 0.05). The inoculated sausages exhibited a significantly lower shear force than the controls (*p* < 0.05). On day 12, the shear force of the PK, TD, and DH treatments reached 16.56, 18.58, and 16.59 N, respectively, which was well below the values observed in the uninoculated CT (26.05 N) and CR (20.57 N) treatments. This was consistent with previous studies that reported that some yeasts could promote the degradation of myofibrillar proteins causing a decrease in shear force, particularly at the end of fermentation [38].

The L*-values of all dry sausages showed a significant reduction after 8 days of fermentation (*p* < 0.05), and except for the PK treatment, the L*-values of all treatments did not change from day 8 to 12 (*p* > 0.05). The decrease in the L*-values may be associated with the aforementioned decrease in the moisture content. The thin aqueous layer on the surface of the meat was disrupted during sausage fermentation, thus, generating less light scattering and darkening the sausages [39]. This may also explain why the DH treatment had the highest L*-values among all treatments on day 12 (*p* < 0.05). Additionally, the a*-value of the PK treatment was significantly higher than that of the DH treatment on day 12 (*p* < 0.05), but it was not different from the controls and the TD treatment *(p* > 0.05). This is inconsistent with the results found in some studies that yeast exert a stabilising effect on reddening [40], which may be related to differences in product types and manufacturing processes [41]. Compared to day 0, the increase in the b*-values of each dry sausage after fermentation of 12 days may be related to lipid oxidation [42]. Concretely, the reaction between lipid oxidation products and amines in phospholipid head groups or the amines in protein may lead to yellow pigmentation [43]. In this study, neither the salt content nor the yeast inoculation affected the b*-value on each fermentation time (*p* > 0.05).

### 3.2. Electronic Nose Analysis

Electronic nose systems are a reliable means for identifying and distinguishing food odours. These devices detect volatile compounds as electronic signals and output them in digital form without having to classify all volatile substances into their individual components [44]. Table 4 illustrates the response value of each sensor upon analysing the dry sausages. Given that there were no significant differences among the five treatments on day 0 (based on the preliminary experiment and data not given), only one treatment (CT-0d) was used as a representative. After the 12-day fermentation period, all treatments exhibited significant increases in the response values of the W1C, W3C, and W5C sensors (*p* < 0.05) compared to those at the beginning of fermentation (day 0). This may be because moisture evaporation in the sausages during the fermentation process resulted in the concentration of volatile compounds. Specifically, fermentation contributes more aroma compounds to dry sausages, such as ammonia, short-chain alkanes, and aromatic compounds, thus increasing the response values. This observation was in accordance with the volatile compound results. Notably, the response values of the W1S sensor of the PK and DH treatments were significantly higher than those of the control, suggesting that *P. kudriavzevii* and *D. hansenii* inoculation could enhance the levels of volatile compounds with methyl groups in the reduced-salt dry sausages.

**Figure 1:**
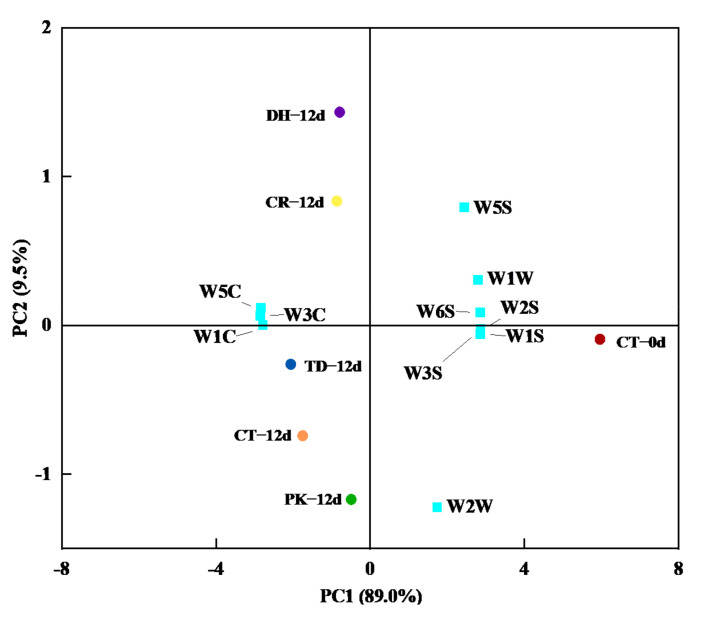
Principal component analysis of electronic nose data for the control and reduced-salt dry sausages with and without yeast inoculation on days 0 and 12. CT: 2.50% NaCl; CR: 1.75% NaCl; PK: 1.75% NaCl + *P. kudriavzevii*; TD: 1.75% NaCl + *T. delbrueckii*; DH: 1.75% NaCl + *D. hansenii*.

The contribution of the first (PC1) and second (PC2) principal components were 89.0% and 9.5%, respectively, thus explaining 98.5% of the total variation (Figure 1). The representative treatment on day 0 (CT-0d) was located in the positive axis of PC1, which was correlated with the W5S, W1W, W6S, W2S, W1S, W3S, and W2W sensors, whereas, all treatments at day 12 were located in the negative axis of PC1, which were correlated with the W5C, W3C, and W1C sensors. The PK and TD treatments were closer to the CT treatment, indicating that the overall odours of the PK and TD treatments were similar to the odour profile of traditional dry sausages.

### 3.3. Electronic Tongue Analysis

**Figure 2:**
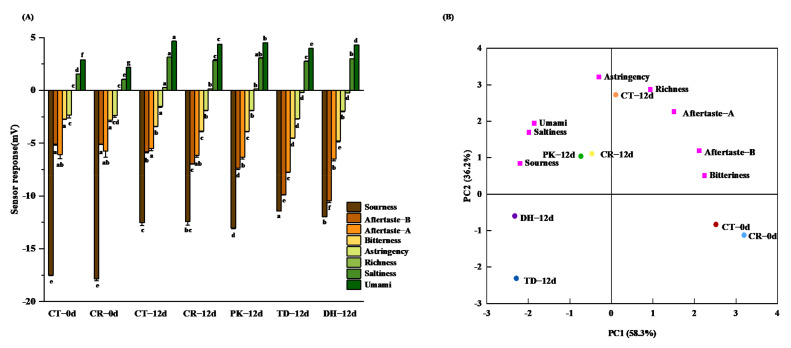
Column chart (**A**) and principal component analysis (**B**) of electronic tongue data for the control and reduced-salt dry sausages with and without yeast inoculation on days 0 and 12. CT: 2.50% NaCl; CR: 1.75% NaCl; PK: 1.75% NaCl + *P. kudriavzevii*; TD: 1.75% NaCl + *T. delbrueckii*; DH: 1.75% NaCl + *D. hansenii*. Different lowercase letters (a–g) for the same taste attribute indicate significant differences among the different treatments (*p* < 0.05).

As illustrated in Figure 2A, the taste properties of dry sausages changed significantly after 12 days of fermentation (*p* < 0.05), compared to those of the CR treatment on day 0, with increases in sourness, umami, and saltiness, and decreases in bitterness, aftertaste-B, and richness. The astringency and aftertaste-A (aftertaste-astringency) remained nearly unchanged compared to those of the CR treatment on day 0. The increase in sourness can be explained by the acid production via the carbohydrate metabolism of LAB in sausages, which was consistent with the pH results [33]. Moreover, the DH treatment had a higher response value for saltiness than the CR treatment, but lower than the CT treatment on day 12 (*p* < 0.05). Further, the saltiness response value of the PK treatment on day 12 reached a similar level to that of the CT treatment on day 12 (*p* > 0.05), indicating that *P. kudriavzevii* and *D. hansenii* could enhance the saltiness of reduced-salt dry sausages. This saltiness enhancement was also observed by Ramos-Moreno et al. [45] when Iberian cured pork loin was inoculated with *D. hansenii*, which was explained by the ability of *D. hansenii* to accumulate high amounts of sodium from the environment without becoming intoxicated [41]. Additionally, the umami response value of the PK treatment on day 12 was higher than that of the CR treatment on day 12, indicating that *P. kudriavzevii* could also enhance umami. This was probably due to the presence of umami amino acids and 5′ nucleotides in yeast strains [46].

PCA was further performed using the data obtained from the electronic tongue analysis of the dry sausages. PC1 and PC2 explained 58.3% and 36.2% of the total variance, respectively, as illustrated in Figure 2B. The CT and CR treatments at day 0 were clustered in the fourth quadrant. After 12 days of fermentation, all treatments were dispersed in the first, second, and third quadrants, indicating that yeast inoculation significantly affected the overall taste profile. On day 12, the CT treatment was associated with astringency, richness, and aftertaste-A, and the PK and CR treatments on day 12 were associated with umami, saltiness, and sourness. Among the inoculated dry sausages, the PK treatment was closer to the CT treatment than the DH and TD treatments, indicating that *P. kudriavzevii* inoculation rendered an overall flavour profile in the reduced-salt sausages that was similar to that of traditional dry sausages. Taken together, our findings indicate that *P. kudriavzevii* inoculation compensated for the lack of saltiness and umami flavours in reduced-salt dry sausages.

### 3.4. Volatile Compound Analysis

As shown in Table 5, 59 volatile compounds were detected in dry sausages, including aldehydes (4), ketones (5), alcohols (12), acids (6), esters (11), terpenes (15), and other compounds (6). These volatile compounds were derived from carbohydrate fermentation and the degradation of free amino acids and fatty acids, thus contributing to the unique flavour profile of dry sausages [47].

Four aldehydes (hexanal, nonanal, tridecanal, and cinnamaldehyde) were identified in the dry sausages. Since hexanal and nonanal are derived from the oxidation of n-6 and n-9 polyunsaturated fatty acid oxidation [48], respectively, they gradually accumulated during the 12-day fermentation period and became the two most abundant aldehydes in the sausages. Hexanal, a typical indicator to evaluate lipid oxidation levels [49] that imparts grass, tallow, and fat flavours to meat products [50], was significantly higher in the PK and DH treatments compared to the controls (*p* < 0.05). Tridecanal, which is linked to floral and sweet flavours, was present in all sausages but its content increased significantly in yeast-inoculated sausages (*p* < 0.05), which is due to lipid oxidation [51].

Ketones are derived from the microbial enzymatic breakdown of lipids or amino acids or by the Maillard reaction [52]. A total of five ketones were detected in the sausages, of which only 3-hydroxy-2-butanone and 2-nonanone were detected in all sausages. 3-Hydroxy-2-butanone is derived from carbohydrate metabolism, whereas 2-nonanone is derived from lipid beta-oxidation. These two methyl ketones are considered characteristic fermented flavour compounds [47]. However, yeast inoculation did not affect the contents of these compounds in the sausages regardless of treatment (*p* > 0.05).

A total of 12 alcohols were detected, which originated from carbohydrate metabolism, reduction of methyl ketones and aldehydes, amino acid metabolism, and lipid oxidation [53,54]. Ethanol, a common alcohol in dry fermented sausages, mainly comes from the wine that is added during sausage preparation. However, this compound can accumulate via the alcoholic fermentation of glucose or the pentose-phosphate route [53]. Ethanol levels remained higher than those of other alcohols throughout the fermentation process but decreased significantly after fermentation (except in the DH treatment), which was likely due to its conversion to esters via interaction with acids [55]. The ethanol content increased significantly in the DH treatment (*p* < 0.05) and the same phenomenon was also confirmed in ‘salchichón’ sausages after inoculation with *D. hansenii* [56], which was likely due to the strong ability of *D. hansenii* to produce alcohols from the metabolism of carbohydrates and branched-chain amino acids [57]. Furthermore, 1-octen-3-ol is an odorant with a mushroom-like flavour that is usually present in meat products [54]. The content of this compound increased significantly after fermentation (*p* < 0.05). On day 12, the PK, TD, and DH treatments exhibited higher content compared to the controls (*p* < 0.05). Further, some alcohols from spices such as linalool and cineole provide dry sausages with characteristic citrus and herbal odours, respectively, and are also important aroma compounds.

Six acids were found in dry sausages, of which acetic and butanoic acids were derived from carbohydrate fermentation, whereas octanoic and nonanoic acids were derived from lipid oxidation [58]. Acids were represented mainly by acetic acid, which can contribute to the typical aroma of meat products, especially dry fermented products [59]. In the present study, the content of acetic acid significantly increased after 12 days of fermentation (*p* < 0.05) and the content of acetic acid was significantly higher in the CR treatment on day 12 (*p* < 0.05), indicating that the lower salt content could promote the production of acetic acid. Among the inoculated sausages, the contents of all acids in the DH treatment were significantly higher than that in the CT and CR treatments (*p* < 0.05). Increases in heptanoic acid and nonanoic acid were found in the PK treatment, and an increase in heptanoic acid was found in the TD treatment (*p* < 0.05). Nevertheless, despite being ester precursors, acids are generally non-essential aroma contributors due to their high odour thresholds.

Esters are crucial contributors to the unique odour profile of dry sausages and generally originate from the non-enzymatic esterification of alcohols and acids, as well as enzymatic catalysis by microorganisms [60]. Most of the esters identified in the present study were ethyl esters, which impart fruity aromas and mask unpleasant odours [48]. These compounds included ethyl acetate, ethyl lactate, ethyl butyrate, ethyl hexanoate, ethyl heptanoate, ethyl caprylate, and ethyl caprate. After 12 days of fermentation, the total ester contents increased in all treatments and each treatment exhibited a unique ester profile. Among the inoculated sausages, the DH treatment showed the highest total ester content at 340.44 μg/kg, followed by the PK and TD treatments, reaching 194.93 and 192.77 μg/kg, respectively, suggesting that yeast inoculation can promote the production of esters that could positively affect the dry sausage aroma. The same phenomenon has been reported in different meat products, such as fermented sausages [56], dry-cured ham [61], and cured pork [46]. However, these effects depend largely on the genus and species of the inoculated yeasts.

A total of 15 terpenes and six other compounds were detected, most of which were not related to chemical or biological processes, but may be related to the spices added during sausage preparation. As a result of the fermentation process, the contents of some compounds [sabinene, γ-terpinene, (1S)-(1)-β-pinene and 3-carene] from spices increased to various degrees.

### 3.5. PLS-DA of Volatile Compounds

**Figure 3:**
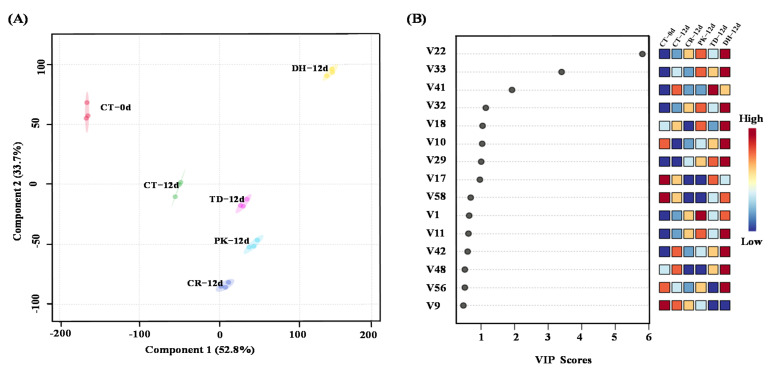
Partial least squares-discriminant analysis score plot (**A**) and the top 15 variables important in the projection (VIP) values (**B**) of all volatile compounds for the control and reduced-salt dry sausages with and without yeast inoculation on days 0 and 12. V9-V58: the numbers of the corresponding volatile compounds in Table 5. CT: 2.50% NaCl; CR: 1.75% NaCl; PK: 1.75% NaCl + *P. kudriavzevii*; TD: 1.75% NaCl + *T. delbrueckii*; DH: 1.75% NaCl + *D. hansenii*.

PLS-DA was performed to explore the differences of the volatiles in reduced-salt sausages inoculated with different yeast strains and to clarify the dynamics changes in these volatiles before and after fermentation. Figure 3A shows the classification pattern of dry sausages by the PLS-DA model with variance explanation 52.8% and 33.7% of component 1 and component 2, respectively. The score plot of the PLS-DA model showed that the six treatments were segregated well from each other. Among them, the CT sausages at days 0 and 12 were clustered on the left side of the score plot, while the CR, PK, TD and DH sausages at day 12 were clustered on the right side of the score plot.

Variable importance in prediction (VIP) can be used to assess the influence strength and explanatory ability of each variable on classification and identification, in which the variable with VIP > 1 is considered to play a significant role [58,59]. Figure 3B showed the 15 top differential volatile compounds, including one acid (acetic acid), two alcohols (linolool and ethanol), three esters (ethyl hexanoate, methyl hexanaoate, and ethyl lactate), and one terpene [(+)-dipentene] with VIP values > 1 were identified as potential classification compounds for the different dry sausages. The higher the VIP value, the greater difference of the aroma compounds between groups, and more important to the discrimination of the aroma types of dry sausages [62]. Notably, inoculation with *D. hansenii* significantly increased the content of six differential compounds [except (+)-dipentene] compared to the CR treatment, while inoculation with *P. kudriavzevii* significantly increased the content of five differential compounds [except (+)-dipentene and ethyl hexanoate]. Inoculation with *T. delbrueckii* only had a significant elevating effect on the content of ethanol, ethyl lactate, and (+)-dipentene compared to the CR treatment, implying that the flavour profiles varied depending on the yeast strains used.

### 3.6. Sensory Evaluation

Table 6 summarizes the results of the sensory evaluation, including colour, hardness, aroma, sourness, saltiness, and umami. There was no significant difference in the colour score (*p* > 0.05); however, this was not consistent with the instrumental colour measurement results, which are more accurate than sensory evaluation. In terms of hardness, the PK treatment score was significantly lower than that of the controls (*p* < 0.05), which was in line with the shear force analysis results. Moreover, the aroma scores of the inoculated treatments were significantly higher than those of the controls (*p* < 0.05). This may be associated with the higher abundance of volatiles detected in the inoculated treatments. In terms of taste, the DH and PK treatments had higher saltiness scores, and the PK treatment presented higher umami scores which were consistent with the electronic tongue analysis results. In contrast, there were no significant differences in the sourness of the dry sausages (*p* > 0.05), which may be because the change in the acidity of the dry sausages was not enough to affect their perceived sourness. Combining these results, we concluded that *D. hansenii* and *P. kudriavzevii* inoculation can compensate for the flavour deficiencies of reduced-salt dry sausages.

Based on the above analysis, it was speculated that the complex microbial enzymes involved in relevant the enzymatic breakdown of carbohydrates, proteins and lipids that affect colour, flavour and texture may be important to improve the quality characteristics of reduced-salt dry sausages. Previous studies have purified a variety of endo- and exo-peptidases from yeast which facilitate the production of FAAs and peptides in meat products [63], which were the flavour compounds and the precursors of flavour compounds. Particularly, some FAAs and peptides can provide saltiness and umami and thus may enhance the taste properties of reduced-salt dry sausages [23]. In addition, a glutaminase that purified from *D. hansenii* was able to neutralize the acid pH of fermented sausages and generated _L_-glutamate that can act as a flavour enhancer [64].

## 4. Conclusions

Our results indicated that yeast inoculation has different effects on the physical, microbial and flavour characteristics of dry sausages. *D. hansenii* inoculation increased the moisture content and L*-value, whereas all three yeast strains reduced hardness. Additionally, both salt reduction and yeast inoculation changed the flavour profiles (taste and odour properties) of dry sausages. Yeast inoculation, particularly *P. kudriavzevii* and *D. hansenii*, compensated for the saltiness and umami of reduced-salt sausages. Furthermore, yeast inoculation promoted the formation of volatile compounds (mainly alcohol, esters, terpenes, and some acids) and enhanced the sensory quality of reduced-salt dry sausages. In conclusion, *P. kudriavzevii* and *D. hansenii* are effective starter cultures that can compensate for the quality and flavour deficiencies of reduced-salt dry sausages.

## Figures and Tables

**Table 1:** The recipes and starter cultures for different sausages.

	CT	CR	PK	TD	DH
Starter cultures	-	-	*P. kudriavzevii*	*T. delbrueckii*	*D. hansenii*
Lean pork (g)	5400	5400	5400	5400	5400
Pork back fat (g)	600	600	600	600	600
Salt (g)	150	105	105	105	105
Sodium nitrite (g)	0.54	0.54	0.54	0.54	0.54
Monosodium glutamate (g)	18	18	18	18	18
Dextrose (g)	20	20	20	20	20
Wine (g)	60	60	60	60	60
Ginger powder (g)	30	30	30	30	30
Mixed spices (g)	48	48	48	48	48

CT: 2.50% NaCl; CR: 1.75% NaCl; PK: 1.75% NaCl + *P. kudriavzevii*; TD: 1.75% NaCl + *T. delbrueckii*; DH: 1.75% NaCl + *D. hansenii.*

**Table 2:** The information on ten electronic nose sensors.

Sensor Name	Representative Substance Type	Description
W1C	Aromatic	Sensitive to aromatic compounds
W5S	Broad range	Sensitive to nitrogen oxides
W3C	Aromatic	Sensitive to aroma, aromatic compounds
W6S	Hydrogen	Sensitive to hydrides
W5C	Arom-aliph	Sensitive to short-chain alkane aromatic compounds
W1S	Broad-methane	Sensitive to methyl
W1W	Sulphur-organic	Sensitive to sulphides
W2S	Broad-alcohol	Sensitive to alcohol, aldehydes and ketones
W2W	Sulph-chlor	Sensitive to organic sulphides
W3S	Methane-aliph	Sensitive to long-chain alkanes

**Table 3:** Changes in physical, microbial, and quality characteristics of the control and reduced-salt dry sausages with and without inoculation of different yeast strains during a 12-day fermentation period.

	Fermentation Time (Day)	CT	CR	PK	TD	DH
Moisture content (%)	0	70.60 ± 2.25 ^Aa^	69.63 ± 3.12 ^Aa^	71.28 ± 3.72 ^Aa^	70.65 ± 1.13 ^Aa^	71.47 ± 0.52 ^Aa^
	4	37.27 ± 1.24 ^Bc^	40.79 ± 0.98 ^Ba^	40.54 ± 0.37 ^Bab^	42.27 ± 0.54 ^Ba^	38.58 ± 0.60 ^Bbc^
	8	22.89 ± 0.28 ^Cc^	25.81 ± 0.16 ^Cb^	25.77 ± 0.70 ^Cb^	25.63 ± 0.25 ^Cb^	27.46 ± 0.19 ^Ca^
	12	18.35 ± 0.04 ^Dc^	19.36 ± 0.32 ^Db^	19.79 ± 0.27 ^Db^	19.85 ± 0.19 ^Db^	21.56 ± 0.44 ^Da^
pH	0	6.10 ± 0.03 ^Aa^	6.09 ± 0.04 ^Aa^	6.08 ± 0.03 ^Aa^	6.09 ± 0.03 ^Aa^	6.09 ± 0.04 ^Aa^
	4	5.69 ± 0.02 ^Ba^	5.55 ± 0.02 ^Bb^	5.56 ± 0.03 ^Bb^	5.28 ± 0.04 ^Bc^	5.56 ± 0.02 ^Bb^
	8	5.59 ± 0.01 ^Ca^	5.40 ± 0.02 ^Cb^	5.16 ± 0.02 ^Cd^	5.07 ± 0.02 ^Ce^	5.31 ± 0.01 ^Cc^
	12	5.65 ± 0.04 ^BCa^	5.45 ± 0.02 ^Cb^	5.20 ± 0.01 ^Cd^	5.12 ± 0.02 ^Ce^	5.34 ± 0.01 ^Cc^
Yeast count (log CFU/g)	0	4.80 ± 0.02 ^Db^	4.80 ± 0.01 ^Cb^	6.12 ± 0.04 ^Ca^	6.08 ± 0.04 ^Da^	6.07 ± 0.04 ^Da^
	4	5.35 ± 0.03 ^Ce^	6.04 ± 0.03 ^Bd^	7.42 ± 0.05 ^Bc^	7.75 ± 0.02 ^Aa^	7.52 ± 0.04 ^Ab^
	8	5.68 ± 0.03 ^Ad^	6.30 ± 0.06 ^Ac^	7.56 ± 0.03 ^Aa^	7.39 ± 0.03 ^Bb^	7.36 ± 0.03 ^Bb^
	12	5.54 ± 0.02 ^Bc^	6.23 ± 0.02 ^Ab^	7.28 ± 0.04 ^Ba^	7.22 ± 0.01 ^Ca^	7.21 ± 0.03 ^Ca^
LAB count (log CFU/g)	0	5.14 ± 0.02 ^Ca^	5.14 ± 0.01 ^Da^	5.15 ± 0.01 ^Da^	5.14 ± 0.01 ^Ca^	5.15 ± 0.01 ^Da^
	4	6.86 ± 0.01 ^Bd^	6.93 ± 0.01 ^Cc^	7.25 ± 0.02 ^Cb^	7.42 ± 0.02 ^Aa^	7.24 ± 0.02 ^Cb^
	8	7.09 ± 0.06 ^Ae^	7.45 ± 0.03 ^Ac^	7.81 ± 0.02 ^Aa^	7.35 ± 0.03 ^Ad^	7.65 ± 0.02 ^Ab^
	12	7.04 ± 0.04 ^Ad^	7.33 ± 0.03 ^Bb^	7.56 ± 0.03 ^Ba^	7.20 ± 0.05 ^Bc^	7.56 ± 0.02 ^Ba^
Shear force (N)	0	3.03 ± 0.18 ^Da^	2.83 ± 0.28 ^Da^	2.82 ± 0.12 ^Da^	2.75 ± 0.41 ^Da^	3.33 ± 0.15 ^Da^
	4	7.62 ± 0.30 ^Ca^	7.62 ± 0.18 ^Ca^	7.60 ± 0.16 ^Ca^	7.55 ± 0.13 ^Ca^	7.54 ± 0.42 ^Ca^
	8	19.56 ± 0.34 ^Ba^	16.61 ± 0.21 ^Bb^	14.54 ± 0.17 ^Be^	15.90 ± 0.14 ^Bc^	15.17 ± 0.13 ^Bd^
	12	26.05 ± 0.79 ^Aa^	20.57 ± 0.26 ^Ab^	16.56 ± 0.34 ^Ad^	18.58 ± 0.21 ^Ac^	16.59 ± 0.18 ^Ad^
L*-value	0	44.54 ± 0.78 ^Aa^	44.17 ± 0.39 ^Aa^	44.53 ± 0.18 ^Aa^	44.16 ± 0.27 ^Aa^	44.40 ± 0.69 ^Aa^
	4	40.52 ± 0.20 ^Ba^	40.46 ± 0.54 ^Ba^	40.21 ± 0.27 ^Ba^	40.39 ± 0.28 ^Ba^	40.61 ± 0.27 ^Ba^
	8	38.34 ± 0.12 ^Cc^	38.52 ± 0.25 ^Cc^	38.72 ± 0.38 ^Cbc^	39.60 ± 0.43 ^Ca^	39.47 ± 0.26 ^Cab^
	12	38.13 ± 0.09 ^Cb^	38.39 ± 0.09 ^Cb^	38.21 ± 0.23 ^Cb^	38.14 ± 0.13 ^Db^	39.12 ± 0.22 ^Ca^
a*-value	0	11.48 ± 0.33 ^Da^	11.63 ± 0.48 ^Ca^	11.60 ± 0.09 ^Da^	11.67 ± 0.07 ^Da^	11.56 ± 0.35 ^Ca^
	4	13.23 ± 0.19 ^Ca^	13.42 ± 0.18 ^Ba^	13.42 ± 0.17 ^Ca^	13.34 ± 0.37 ^Ca^	13.33 ± 0.22 ^Ba^
	8	14.20 ± 0.05 ^Bb^	14.15 ± 0.20 ^Bb^	14.82 ± 0.21 ^Ba^	14.28 ± 0.27 ^Bb^	14.23 ± 0.19 ^Ab^
	12	14.89 ± 0.14 ^Aab^	15.00 ± 0.18 ^Aab^	15.41 ± 0.02 ^Aa^	15.01 ± 0.29 ^Aab^	14.82 ± 0.24 ^Ab^
b*-value	0	15.37 ± 0.17 ^Ca^	15.33 ± 0.12 ^Ca^	15.31 ± 0.21 ^Ca^	15.39 ± 0.13 ^Ca^	15.34 ± 0.11 ^Ca^
	4	17.46 ± 0.51 ^Ba^	17.38 ± 0.34 ^Ba^	17.22 ± 0.23 ^Ba^	17.36 ± 0.39 ^Ba^	17.42 ± 0.28 ^Ba^
	8	17.62 ± 0.10 ^Aa^	17.78 ± 0.14 ^Ba^	17.53 ± 0.23 ^Ba^	17.48 ± 0.18 ^Ba^	17.75 ± 0.16 ^Ba^
	12	18.32 ± 0.14 ^Aa^	18.39 ± 0.14 ^Aa^	18.37 ± 0.17 ^Aa^	18.44 ± 0.17 ^Aa^	18.46 ± 0.29 ^Aa^

Different uppercase letters (A–D) within a column for the same indicator indicate significant differences among the different fermentation days (*p* < 0.05). Different lowercase letters (a–e) within a row for the same fermentation time indicate significant differences among the different treatments (*p* < 0.05). CT: 2.50% NaCl; CR: 1.75% NaCl; PK: 1.75% NaCl + *P. kudriavzevii*; TD: 1.75% NaCl + *T. delbrueckii*; DH: 1.75% NaCl + *D. hansenii*.

**Table 4:** Response values of electronic nose sensors of the control and reduced-salt dry sausages with and without yeast inoculation on days 0 and 12.

Sensor Name	CT-0d	CT-12d	CR-12d	PK-12d	TD-12d	DH-12d
W1C	0.14 ± 0.04 b	0.33 ± 0.07 ^a^	0.30 ± 0.06 ^a^	0.31 ± 0.02 ^a^	0.39 ± 0.05 ^a^	0.33 ± 0.02 ^a^
W5S	2.11 ± 0.13 ^a^	1.69 ± 0.13 ^b^	1.88 ± 0.18 ^ab^	1.76 ± 0.08 ^ab^	1.73 ± 0.20 ^ab^	1.95 ± 0.11 ^ab^
W3C	0.18 ± 0.02 c	0.41 ± 0.03 ^ab^	0.38 ± 0.02 ^ab^	0.35 ± 0.02 ^b^	0.43 ± 0.04 ^a^	0.38 ± 0.01 ^ab^
W6S	8.67 ± 2.65 ^a^	3.67 ± 0.68 ^b^	4.22 ± 0.72 ^b^	3.90 ± 0.24 ^b^	3.47 ± 0.47 ^b^	4.20 ± 0.27 ^b^
W5C	0.24 ± 0.03 ^c^	0.58 ± 0.03 ^a^	0.53 ± 0.03 ^ab^	0.47 ± 0.03 ^b^	0.58 ± 0.04 ^a^	0.53 ± 0.01 ^ab^
W1S	79.95 ± 0.12 ^a^	20.70 ± 0.64 ^e^	22.53 ± 0.19 ^d^	27.03 ± 0.13 ^b^	19.56 ± 0.29 ^f^	25.50 ± 0.16 ^c^
W1W	2.42 ± 0.25 ^ab^	1.72 ± 0.11 ^c^	1.84 ± 0.12 ^c^	1.78 ± 0.07 ^c^	1.77 ± 0.10 ^c^	1.93 ± 0.06 ^bc^
W2S	13.18 ± 4.06 ^a^	4.56 ± 0.73 ^b^	5.13 ± 0.71 ^b^	5.26 ± 0.48 ^b^	4.09 ± 0.5 ^b^	4.87 ± 0.24 ^b^
W2W	0.76 ± 0.02 ^a^	0.73 ± 0.02 ^abc^	0.70 ± 0.01 ^bc^	0.75 ± 0.02 ^ab^	0.72 ± 0.01 ^abc^	0.69 ± 0.01 ^c^
W3S	3.40 ± 0.31 ^a^	1.62 ± 0.07 ^b^	1.80 ± 0.10 ^b^	1.88 ± 0.08 ^b^	1.68 ± 0.10 ^b^	1.82 ± 0.06 ^b^

Different lowercase letters (a–f) within a row indicate significant differences among the different treatments (*p* < 0.05). CT: 2.50% NaCl; CR: 1.75% NaCl; PK: 1.75% NaCl + *P. kudriavzevii*; TD: 1.75% NaCl + *T. delbrueckii*; DH: 1.75% NaCl + *D. hansenii*.

**Table 5:** Volatile compounds identified and quantified (μg/kg) by gas chromatography/mass spectrometry (GC-MS) of the control and reduced-salt dry sausages with and without yeast inoculation on days 0 and 12.

	Volatile Compound	CAS	LRI	CT-0d	CT-12d	CR-12d	PK-12d	TD-12d	DH-12d
	Aldehydes								
V1	Hexanal	66-25-1	1084	n.d.	8.58 ± 0.30 ^c^	12.03 ± 1.61 ^c^	35.79 ± 2.19 ^a^	11.93 ± 0.38 ^c^	25.05 ± 0.85 ^b^
V2	Nonanal	124-19-6	1385	1.43 ± 0.08 ^e^	10.50 ± 0.54 ^c^	16.25 ± 1.40 ^b^	20.83 ± 0.55 ^a^	11.31 ± 0.45 ^c^	7.74 ± 0.55 ^d^
V3	Tridecanal	10486-19-8	1767	1.39 ± 0.11 ^e^	2.04 ± 0.06 ^d^	2.07 ± 0.10 ^d^	3.77 ± 0.16 ^b^	3.02 ± 0.18 ^c^	4.89 ± 0.20 ^a^
V4	Cinnamaldehyde	104-55-2	2170	9.48 ± 0.51 ^a^	3.52 ± 0.10 ^d^	2.08 ± 0.06 ^e^	4.41 ± 0.13 ^c^	2.57 ± 0.06 ^e^	6.41 ± 0.14 ^b^
	Total			12.30 ± 0.18 ^f^	24.64 ± 0.16 ^e^	32.43 ± 0.33 ^c^	64.80 ± 0.74 ^a^	28.83 ± 0.34 ^d^	44.09 ± 0.52 ^b^
	Ketones								
V5	3-Hydroxy-2-butanone	513-86-0	1299	3.86 ± 0.10 ^b^	24.29 ± 1.81 ^a^	23.96 ± 0.78 ^a^	23.19± 0.33 ^a^	24.75 ± 0.30 ^a^	24.41 ± 0.85 ^a^
V6	2-Nonanone	821-55-6	1396	5.78 ± 0.30 ^a^	4.19 ± 0.23 ^c^	3.30 ± 0.18 ^d^	4.11 ± 0.27 ^c^	5.02 ± 0.33 ^b^	4.16 ± 0.21 ^c^
V7	2,3-Pentanedione	600-14-6	1120	n.d.	n.d.	n.d.	1.79 ± 0.09 ^a^	n.d.	n.d.
V8	1-Octen-3-one	4312-99-6	1283	n.d.	n.d.	n.d.	1.43 ± 0.04 ^b^	1.57 ± 0.06 ^b^	2.09 ± 0.07 ^a^
V9	6-Methyl-5-hepten-2-one	110-93-0	1544	17.20 ± 0.66 ^a^	9.47 ± 0.45 ^b^	5.59 ± 0.14 ^c^	3.82 ± 0.16 ^d^	n.d.	n.d.
	Total			26.84 ± 0.71 ^c^	37.95 ± 2.21 ^a^	32.85 ± 1.19 ^b^	34.34 ± 0.45 ^ab^	31.34 ± 0.79 ^bc^	30.66 ± 1.17 ^bc^
	Alcohols								
V10	Ethanol	64-17-5	928	109.89 ± 4.30 ^b^	67.16 ± 0.95 ^e^	69.72 ± 1.15 ^e^	80.14 ± 1.23 ^d^	88.62 ± 2.43 ^c^	143.23 ± 3.45 ^a^
V11	2,3-Butanediol	513-85-9	1590	n.d.	10.96 ± 0.38 ^c^	24.50 ±1.16 ^a^	25.29 ± 1.61 ^a^	17.83 ± 1.53 ^b^	26.96 ± 1.92 ^a^
V12	1-Octen-3-ol	3391-86-4	1451	0.77 ± 0.06 ^e^	3.10 ± 0.16 ^d^	3.15 ± 0.16 ^d^	7.84 ± 0.38 ^a^	4.66 ± 0.20 ^c^	6.82 ± 0.41 ^b^
V13	2-Heptanol	543-49-7	1285	2.30 ± 0.16 ^bc^	2.07 ± 0.23 ^cd^	1.59 ± 0.08 ^e^	2.52 ± 0.07 ^b^	1.87 ± 0.04 ^de^	3.05 ± 0.14 ^a^
V14	3-Phenyl-1-propanol	122-97-4	1715	6.00 ± 0.21 ^a^	n.d.	n.d.	n.d.	n.d.	n.d.
V15	Benzyl alcohol	100-51-6	1618	n.d.	5.58 ± 0.24 ^a^	3.18 ± 0.13 ^c^	3.78 ± 0.11 ^b^	5.46 ± 0.20 ^a^	3.51 ± 0.08 ^bc^
V16	Decyl alcohol	112-30-1	1300	n.d.	n.d.	n.d.	n.d.	n.d.	14.39 ± 1.37 ^a^
V17	Cineole	470-82-6	1224	120.64 ± 4.65 ^a^	82.64 ± 2.28 ^c^	66.79 ± 1.92 ^d^	92.12 ± 3.86 ^b^	83.14 ± 2.94 ^bc^	75.24 ± 3.69 ^cd^
V18	Linalool	78-70-6	1552	117.24 ± 3.41 ^bc^	119.31 ± 3.82 ^b^	101.63 ± 4.92 ^d^	126.70 ± 3.28 ^b^	107.17 ± 3.35 ^cd^	168.38 ± 3.78 ^a^
V19	Geraniol	106-24-1	1849	5.12 ± 0.16 ^bc^	8.99 ± 0.83 ^a^	5.25 ± 0.69 ^bc^	4.07 ± 0.31 ^c^	4.00 ± 0.25 ^c^	6.44 ± 0.51 ^b^
V20	(-)-α-Terpineol	10482-56-1	1517	22.59 ± 1.32 ^a^	12.92 ± 0.69 ^c^	9.91 ± 0.44 ^d^	12.48 ± 0.75 ^c^	11.60 ± 0.57 ^cd^	16.21 ± 1.16 ^b^
V21	(-)-Terpinen-4-ol	20126-76-5	1466	44.14 ± 2.09 ^b^	37.81 ± 1.71 ^cd^	30.07 ± 0.98 ^e^	40.04 ± 1.19 b^c^	35.83 ± 1.34 ^d^	51.78 ± 1.43 ^a^
	Total			428.69 ± 4.78 ^b^	350.54 ± 6.29 ^d^	315.79 ± 5.89 ^e^	394.98 ± 6.75 ^c^	360.18 ± 4.21 ^d^	516.01 ± 7.81 ^a^
	Acids								
V22	Acetic acid	64-19-7	1450	5.49 ± 0.48 ^e^	90.27 ± 1.81 ^d^	183.34 ± 4.85 ^bc^	191.23 ± 5.63 ^b^	171.00 ± 5.32 ^c^	227.66 ± 5.94 ^a^
V23	Isovaleric acid	503-74-2	1665	2.48 ± 0.18 ^c^	5.15 ± 0.41 ^b^	5.39 ± 0.35 ^b^	6.37 ± 0.81 ^b^	6.41 ± 0.76 ^b^	9.23 ± 0.88 ^a^
V24	Heptanoic acid	111-14-8	2168	n.d.	2.22 ± 0.33 ^c^	1.24 ± 0.16 ^c^	4.82 ± 0.57 ^b^	6.26 ± 0.64 ^b^	15.06 ± 0.92 ^a^
V25	Nonanoic acid	112-05-0	2202	0.88 ± 0.10 ^c^	1.28 ± 0.18 ^c^	1.30 ± 0.13 ^c^	2.94 ± 0.20 ^b^	0.92 ± 0.07 ^c^	3.73 ± 0.23 ^a^
V26	Butanoic acid	107-92-6	1477	3.62 ± 0.18 ^e^	4.92 ± 0.42 ^de^	6.80 ± 0.25 ^bc^	8.53 ± 0.61 ^b^	6.41 ± 0.82 ^cd^	12.18 ± 1.17 ^a^
V27	Octanoic acid	124-07-2	2083	n.d.	8.57 ± 0.45 ^b^	8.18 ± 0.71 ^b^	8.61 ± 0.76 ^b^	10.11 ± 0.86 ^b^	18.75 ±1.19 ^a^
	Total			12.47 ± 0.37 ^e^	112.41 ± 4.30 ^d^	206.25 ± 6.01 b^c^	222.50 ± 6.82 ^b^	201.11 ± 3.63 ^c^	286.61 ± 7.45 ^a^
	Esters								
V28	Ethyl acetate	141-78-6	907	1.21 ± 0.13 ^d^	4.07 ± 0.25 ^d^	18.45 ± 0.93 ^b^	16.34 ± 0.99 ^b^	23.12 ±1.12 ^a^	11.87 ± 0.66 ^c^
V29	Ethyl lactate	97-64-3	1358	n.d.	n.d.	5.13 ± 0.44 ^c^	20.27 ± 0.76 ^b^	21.42 ± 0.85 ^b^	32.52 ± 0.61 ^a^
V30	Methyl butyrate	623-42-7	963	n.d.	n.d.	n.d.	7.84 ± 0.52 ^b^	1.05 ± 0.08 ^c^	9.31 ± 0.68 ^a^
V31	Ethyl butyrate	105-54-4	1028	n.d.	2.92 ± 0.35 ^c^	3.94 ± 0.44 ^bc^	4.53 ± 0.28 ^b^	4.26 ± 0.40 ^bc^	12.14 ± 0.88 ^a^
V32	Methyl hexanoate	106-70-7	1188	13.66 ± 0.59 ^d^	25.62 ± 0.99 ^c^	39.92 ± 1.34 ^b^	43.47 ±1.63 ^b^	28.98 ± 1.46 ^c^	64.29 ± 2.26 ^a^
V33	Ethyl hexanoate	123-66-0	1120	18.71 ± 0.72 ^e^	51.85 ± 2.29 ^c^	42.34 ± 1.34 ^d^	74.67 ± 1.43 ^b^	72.45 ± 1.67 ^b^	159.18 ± 3.78 ^a^
V34	Ethyl heptanoate	106-30-9	1328	1.08 ± 0.17 ^b^	3.30 ± 0.37 ^a^	2.71 ± 0.38 ^a^	n.d.	3.01 ± 0.42 ^a^	n.d.
V35	Ethyl caprylate	106-32-1	1437	1.43 ± 0.23 ^d^	6.59 ± 0.54 ^c^	7.59 ± 0.61 ^c^	10.08 ± 0.82 ^b^	11.36 ± 0.59 ^b^	20.04 ± 1.00 ^a^
V36	Bornyl acetate	76-49-3	1288	10.92 ± 0.41 ^c^	12.30 ± 0.48 ^abc^	9.09 ± 0.59 ^d^	11.57 ± 0.64 ^bc^	13.53 ± 0.57 ^a^	13.19 ± 0.93 ^ab^
V37	Ethyl caprate	110-38-3	1634	n.d.	5.18 ± 0.47 ^c^	5.73 ± 0.55 ^c^	6.16 ± 0.50 ^c^	8.40 ± 0.96 ^b^	11.22 ± 0.96 ^a^
V38	Methyl lactate	547-64-8	1293	n.d.	0.96 ± 0.08 ^c^	4.14 ± 0.44 ^b^	n.d.	5.19 ± 0.31 ^b^	6.68 ± 0.72 ^a^
	Total			47.01 ± 1.74 ^e^	112.79 ± 3.49 ^d^	139.04 ± 0.98 ^c^	194.93 ± 3.56 ^b^	192.77 ± 2.96 ^b^	340.44 ± 5.98 ^a^
	Terpenes								
V39	Sabinene	3387-41-5	1160	2.84 ± 0.52 ^c^	11.25 ± 0.92 ^b^	8.43 ± 0.95 ^b^	10.71 ± 1.13 ^b^	11.02 ± 0.86 ^b^	15.31 ± 0.93 ^a^
V40	Myrcene	123-35-3	1145	8.38 ± 0.75 ^b^	12.34 ± 0.93 ^a^	7.84 ± 0.98 ^b^	9.31 ± 1.17 ^b^	12.09 ± 1.09 ^a^	12.17 ± 0.95 ^a^
V41	(+)-Dipentene	5989-27-5	1203	76.70 ± 3.11 ^e^	174.82 ± 6.01 ^ab^	126.56 ± 5.16 ^d^	152.97 ± 4.27 ^c^	181.89 ± 4.51 ^a^	162.42 ± 4.45 b^c^
V42	γ-Terpinene	99-85-4	1178	15.05 ± 0.54 ^f^	35.65 ± 0.62 ^b^	22.08 ± 0.27 ^e^	25.18 ± 0.33 ^d^	34.10 ± 0.44 ^c^	43.34 ± 0.54 ^a^
V43	α-Caryophyllene	6753-98-6	2209	8.66 ± 0.51 ^bc^	3.12 ± 0.31 ^e^	7.04 ± 0.59 ^d^	7.73 ± 0.55 ^cd^	10.36 ± 0.79 ^a^	10.04 ± 0.30 ^ab^
V44	α-Curcumene	644-30-4	1773	80.18 ± 3.47 ^a^	63.90 ± 1.53 ^b^	40.78 ± 0.76 ^c^	45.22 ± 1.10 ^c^	60.45 ± 1.98 ^b^	64.78 ± 1.46 ^b^
V45	α-Farnesene	502-61-4	1543	18.50 ± 0.79 ^a^	14.98 ± 0.50 ^b^	9.44 ± 0.81 ^c^	9.43 ± 0.61 ^c^	14.38 ±0.83 ^b^	14.33 ± 0.88 ^b^
V46	α-Terpinene	99-86-5	1282	4.21 ± 0.44 ^b^	6.12 ± 0.55 ^a^	4.50 ± 0.68 ^b^	4.13 ± 0.25 ^b^	5.24 ± 0.72 ^ab^	4.51 ± 0.68 ^b^
V47	β-Bisabolene	495-61-4	1536	23.70 ± 1.95 ^a^	17.96 ± 0.81 ^b^	11.52 ± 0.60 ^c^	11.99 ± 0.45 ^c^	17.52 ± 0.91 ^b^	17.33 ± 0.83 ^b^
V48	β-Caryophyllene	87-44-5	1594	57.39 ± 1.61 ^c^	82.40 ± 2.28 ^a^	54.82 ± 1.54 ^c^	65.7 ± 1.70 ^b^	79.08 ± 1.34 ^a^	83.45 ± 1.60 ^a^
V49	β-Famesene	18794-84-8	1661	2.29 ± 0.20 ^d^	2.51 ± 0.51 ^d^	3.19 ± 0.14 ^cd^	4.58 ± 0.45 ^b^	3.78 ± 0.35 b^c^	6.62± 0.55 ^a^
V50	(1 S)-(1)-β-Pinene	18172-67-3	1152	1.83 ± 0.18 ^d^	5.20 ± 0.17 ^b^	3.76 ± 0.47 ^c^	5.10 ± 0.25 ^b^	5.22 ± 0.23 ^b^	6.36 ± 0.34 ^a^
V51	3-Carene	13466-78-9	1146	1.32 ± 0.18 ^c^	3.09 ± 0.27 ^ab^	2.51 ± 0.25 ^b^	3.12 ± 0.40 ^ab^	3.61 ± 0.21 ^a^	3.09 ± 0.28 ^ab^
V52	Camphene	79-92-5	1705	9.35 ± 0.65 ^a^	4.00 ± 0.51 ^b^	3.20 ± 0.27 ^b^	3.59 ± 0.21 ^b^	4.10 ± 0.42 ^b^	9.99 ± 1.12 ^a^
V53	Cyclosativene	22469-52-9	1373	3.14 ± 0.23 ^b^	3.75 ± 0.34 ^b^	3.05 ± 0.52 ^b^	3.93 ± 0.40 ^b^	4.08 ± 0.45 ^b^	5.42 ± 0.62 ^a^
	Total			325.40 ± 5.15 ^c^	453.54 ± 7.54 ^a^	316.05 ± 2.63 ^c^	407.91 ± 5.97 ^b^	457.38 ± 6.22 ^a^	470.86 ± 7.16 ^a^
	Others								
V54	D-Camphor	464-49-3	1427	12.34 ± 0.24 f	17.03 ± 0.20 ^d^	15.33 ± 0.41 ^e^	25.08 ± 0.61 ^b^	18.97 ± 0.54 ^c^	27.64 ±0.34 ^a^
V55	4-Allylanisole	140-67-0	1655	164.56 ± 4.45 ^b^	161.06 ± 4.14 ^b^	108.28 ± 2.45 ^d^	129.21 ± 2.56 ^d^	146.72 ± 2.23 ^c^	188.07 ± 4.21 ^a^
V56	Anethole	104-46-1	1595	315.01 ± 11.00 ^b^	246.66 ± 8.66 ^d^	232.42 ± 7.37 ^d^	275.28 ± 5.57 ^c^	224.05 ± 6.32 ^e^	344.05 ± 5.87 ^a^
V57	Safrole	94-59-7	1628	12.59 ± 0.51 ^bc^	14.59± 0.45 ^ab^	8.91 ± 0.68 ^d^	10.48 ± 0.75 ^cd^	13.99 ± 0.65 ^ab^	15.97 ± 1.34 ^a^
V58	Eugenol	97-53-0	2141	240.48 ± 6.94 ^a^	187.47 ± 5.33 ^b^	136.75 ± 5.46 ^c^	140.72 ± 4.47 ^c^	148.83 ± 5.12 ^c^	231.42 ± 6.27 ^a^
V59	Methyl eugenol	93-15-2	1755	5.89 ± 0.24 ^c^	8.00 ± 0.37 ^b^	2.49 ± 0.08 ^d^	3.38 ± 0.13 ^d^	6.78 ± 0.34 ^c^	14.27 ± 0.55 ^a^
	Total			750.87 ± 10.83 ^b^	634.81 ± 19.57 ^c^	504.18 ± 6.86 ^e^	584.15 ± 5.57 ^d^	559.34 ± 7.57 ^d^	821.42 ± 12.30 ^a^

Different lowercase letters (a–f) within the same row indicate significant differences among the different treatments (*p* < 0.05). n.d.: not detected. LRI: linear retention index. CAS: the unique numerical identification number for substance specified by the Chemical Abstracts Service. CT: 2.50% NaCl; CR: 1.75% NaCl; PK: 1.75% NaCl + *P. kudriavzevii*; TD: 1.75% NaCl + *T. delbrueckii*; DH: 1.75% NaCl + *D. hansenii*.

**Table 6:** Sensory evaluation of the control and reduced-salt dry sausages with and without yeast inoculation after a 12-day fermentation period.

Attribute	CT	CR	PK	TD	DH
Colour	5.52 ± 0.33 ^a^	5.15 ± 0.26 ^a^	5.32 ± 0.43 ^a^	5.18 ± 0.33 ^a^	5.48 ± 0.38 ^a^
Hardness	6.23 ± 0.42 ^a^	5.53 ± 0.35 ^ab^	4.33 ± 0.45 ^c^	5.47 ± 0.31 ^ab^	4.83 ± 0.15 ^bc^
Amora	4.83 ± 0.25 ^b^	4.80 ± 0.46 ^b^	5.73 ± 0.31 ^a^	5.97 ± 0.25 ^a^	6.30 ± 0.20 ^a^
Sourness	4.37 ± 0.40 ^a^	4.60 ± 0.26 ^a^	4.43 ± 0.25 ^a^	4.40 ± 0.30 ^a^	4.47 ± 0.21 ^a^
Saltiness	5.60 ±0.30 ^a^	3.27 ± 0.31 ^c^	4.20 ± 0.26 ^b^	3.33 ± 0.32 ^c^	4.40 ± 0.26 ^b^
Umami	5.07 ± 0.35 ^a^	4.60 ± 0.26 ^ab^	4.97 ± 0.31 ^a^	4.33 ± 0.25 ^ab^	4.07 ± 0.25 ^b^

Different lowercase letters (a–c) within a row indicate significant differences among the different treatments (*p* < 0.05). CT: 2.50% NaCl; CR: 1.75% NaCl; PK: 1.75% NaCl + *P. kudriavzevii*; TD: 1.75% NaCl + *T. delbrueckii*; DH: 1.75% NaCl + *D. hansenii*.

## Data Availability

The data presented in this study are available within the article.
